# Elevated High-Sensitivity C-Reactive Protein Levels Predict Decreased Survival for Nasopharyngeal Carcinoma Patients in the Intensity-Modulated Radiotherapy Era

**DOI:** 10.1371/journal.pone.0122965

**Published:** 2015-04-13

**Authors:** Lin Quan Tang, Dong Peng Hu, Qiu Yan Chen, Lu Zhang, Xiao Ping Lai, Yun He, Yun-Xiu-Xiu Xu, Shi-Hua Wen, Yu-Tuan Peng, Wen-Hui Chen, Shan-Shan Guo, Li-Ting Liu, Chao-Nan Qian, Xiang Guo, Mu-Sheng Zeng, Hai-Qiang Mai

**Affiliations:** 1 State Key Laboratory of Oncology in South China and Collaborative Innovation Center for Cancer Medicine, Sun Yat-sen University Cancer Center, Guangzhou, Guangdong, China; 2 Department of Nasopharyngeal Carcinoma, Sun Yat-sen University Cancer Center, Guangzhou, Guangdong, China; 3 ZhongShan School of Medicine, Sun Yat-sen University, Guangzhou, Guangdong, China; National Cancer Centre, SINGAPORE

## Abstract

**Purpose:**

This study aimed to clarify the prognostic utility of high-sensitivity C-reactive protein (hs-CRP) in nasopharyngeal carcinoma (NPC) patients in the Intensity-Modulated Radiotherapy (IMRT) era.

**Patients and Methods:**

In this observational study, 1,589 non-metastatic NPC patients treated with IMRT were recruited. Blood samples were collected before treatment for examination of hs-CRP levels. We evaluated the association of pretreatment hs-CRP levels with overall survival rate (OS), progression free survival rate (PFS), locoregional relapse free survival rate (LRFS) and distant metastasis free survival rate (DMFS).

**Results:**

Baseline hs-CRP levels were correlated with sex, clinical stage, body mass index, smoking status, and EBV DNA level. Multivariate analysis showed that hs-CRP had significant association with OS (HR:1.723; 95%CI:1.238–2.398; p = 0.001), PFS (HR:1.621; 95%CI:1.273–2.064; p<0.001) and DMFS (HR:1.879; 95%CI:1.394–2.531; p<0.001). In subgroups such as advanced-stage group, low EBV DNA group and high EBV DNA group, elevated hs-CRP levels still predicted poor clinical outcomes. Furthermore, in patients with chronic HBV infection, decreased 4-year survival was observed in the cohort of high hs-CRP levels, with 87.4% vs. 94.9% (p = 0.023) for OS, 65.2% vs. 90.8% (p<0.001) for PFS, and 67.6% vs. 95.0% (p<0.001) for DMFS. A similar finding was observed for patients with cardiovascular disease, with 79.1% vs. 90.2% (p = 0.020) for PFS, and 71.4% vs. 97.6% (p = 0.002) for DMFS.

**Conclusion:**

Elevated serum hs-CRP levels were correlated with poor survival for NPC patients in the IMRT era, playing a complementary role to TNM stage and EBV DNA. In addition, elevated hs-CRP level was still an effective indicator for patients with chronic HBV infection and cardiovascular disease.

## Introduction

Nasopharyngeal carcinoma (NPC) is a head and neck neoplasm of high malignancy, with an extremely skewed distribution across the world. Being endemic in Southeast Asia, it has brought obvious devastation to societies because the peak incidence is at 40 to 50 years of age. Radiotherapy with or without chemotherapy is the primary treatment modality for NPC patients, and 5-year overall survival exceeding 75% can be achieved today [1]. Recently, great achievements in biomarker’s finding and improved treatment methods were successively reported in the battle against NPC, such as plasma Epstein-Barr virus DNA [2] and serum lactate dehydrogenase (LDH) [3], having been identified to be of significant value in refining treatment strategies and predicting outcomes. In particular, plasma EBV DNA has been considered to be a marvelous indicator for the diagnosis, risk stratification, monitoring and prediction of the prognosis of NPC [4–8], and it has been gradually implemented in clinical practice since 2004 when its excellent prognostic value for NPC patients was discovered [7].

In the 21st century, the diagnostic and treatment allocation for NPC has undergone tremendous changes. Intensity-modulated radiotherapy (IMRT) has gradually replaced two-dimensional conventional radiotherapy (2D-CRT) as the primary means of radiotherapy, gaining superior locoregional control [9] and improved long-term survival for patients with NPC [10]. Therefore, it is of interest to determine whether prognostic factors previously evaluated for 2D-CRT can also be applied to modern IMRT. We hypothesize that some extra biomarkers may be complementary to EBV DNA in IMRT.

High-sensitivity C-reactive protein (hs-CRP), an acute phase protein synthesized by the liver, was demonstrated to be correlated with a poorer prognosis in colorectal cancer [11], osteosarcoma [12], hepatocellular carcinoma [13] and other cancers [14], as well as for NPC patients [15]. Besides, previous studies have also proved that patients with cardiovascular disease and chronic hepatitis display increased serum hs-CRP levels [16, 17]. It was perceived that CRP could deposit in arterial intima and recruit monocytes during atherogenesis [18]. While in chronic hepatitis, HBV may enhance the expression of IL-6 [19] and IL-6 would in turn promote the production of CRP [20].

However, findings from previous reports [15] showing the relationship between elevated CRP levels and poor survival for NPC patients were based on two-dimensional radiotherapy (2D-CRT) with a moderate sample size and without adjusting for other factors such as body mass index (BMI) [21], concurrent disease, and smoking status. The prognostic role of baseline hs-CRP levels in patients with NPC treated with IMRT remains unknown.

Therefore, we conducted a large-scale cohort study aimed at examining the role of hs-CRP in prognosis for NPC patients treated with IMRT. Subgroup analyses were also performed in low EBV DNA and high EBV DNA subgroups and in patients with or without comorbidities of cardiovascular disease and chronic HBV infection, evaluating whether hs-CRP still had prognostic value or not when confined to groups mentioned above.

## Patients and Methods

### Patients

NPC patients treated with IMRT were consecutively recruited from Jan 2007 to Dec 2010. Patients were excluded if they met the following criteria: (1) previously received any anticancer therapy; (2) <18 years old; (3) pregnant or lactating; (4) unsuitable for chemotherapy as a result of a liver, kidney, lung, or heart deficiency; (5) a history of previous or synchronous malignant tumors; (6) NPC patients with primary metastasis; or (7) lost during follow-up.

The routine staging work-up included clinical examination of the head and neck region, a magnetic resonance imaging scan from the suprasellar cistern to the collarbone, fiberoptic nasopharyngoscopy, chest radiography, and abdominal sonography and a whole-body bone scan or whole-body FDG PET/CT. All the patients were restaged according to the seventh American Joint Committee on Cancer (AJCC) TNM staging manual. The technique of IMRT has been described in detail [22], and relevant assessments have been continuously reported in anticancer therapy [23, 24].

### Methods

This study was approved by the independent Institute Research Ethics Committee at the Sun Yat-sen University Cancer Center (SYSUCC, Guangzhou, P. R. China), and written consents were obtained from all participants. Before treatment, the following baseline information was collected: sex, age, hereditary NPC, smoking status, and BMI. Information on concurrent diseases, such as cardiovascular disease, diabetes mellitus, or chronic HBV infection, was also collected as previous studies have indicated that these factors may promote increased serum CRP levels [16, 17]. These comorbidities and smoking status were defined as follows: Chronic hepatitis B: HBsAg-positive>6 months and serum HBV-DNA≥2000 IU/ml (10^4^ copies/mL) with or without elevated alanine transaminase/aspartatetransaminase levels; diabetes: fasting plasma glucose level 7.0 mmol/L and/or 2 h plasma glucose level 11.1 mmol/L after a 75 g glucose load or a previous diagnosis of diabetes by a healthcare professional; cardiovascular disease: includes coronary heart disease, cerebro-vascular disease, peripheral arterial disease, rheumatic heart disease, congenital heart disease, deep vein thrombosis, pulmonary embolism, hypertension (systolic blood pressure140 mmHg, diastolic blood pressure 90 mmHg) or a previous diagnosis of any of these diseases by a healthcare professional; smoking status: patients were identified as current, former, or never smokers. Patients who smoked or reported smoking cessation within 1 year of the diagnosis were considered current smokers. Patients who had smoked less than 100 cigarettes during their lifetime were considered never smokers.

### Hs-CRP and EBV DNA measurement

A 3 mL fasting blood sample was collected before treatment. The sample was processed within 3 hours of collection, and serum was stored at -70 to -80°C until analysis. Hs-CRP was determined with immune-turbidometric assay using a HITACHI Automatic Analyzer LABOSPECT008 (Hitachi High-Technologies Corporation, Tokyo, Japan)[25]. Interassay coefficients of variation were less than 5% for hs-CRP. Plasma EBV DNA concentrations were routinely measured using q-PCR before treatment. EBV-specific VCA/IgA antibodies and EBV-specific EA/IgA antibodies were measured using an immunoenzymic assay described previously [26].

### Statistical analysis

Our primary endpoint was overall survival (OS). Progression-free survival (PFS), locoregional relapse-free survival (LRFS) and distant metastasis-free survival (DMFS) were included as secondary endpoints in this article. OS was calculated from the date of the first NPC diagnosis to the date of death from any cause or patient censoring at the date of the last follow-up. PFS was calculated from the date of the first NPC diagnosis to the date of relapse at any site or patient censoring at the date of the last follow-up. LRFS was calculated from the date of the first NPC diagnosis to the date of relapse at head and neck region or patient censoring at the date of the last follow-up. DMFS was determined from the date of the first NPC diagnosis to the date of distant relapse or patient censoring at the date of the last follow-up.

Patients who were still alive as of December of 2013 (end of follow-up) and those who were lost to follow up were censored at the date of the last contact. After treatment was completed, the patients were evaluated at 3-month intervals for the first 3 years and every 6 months thereafter.

All statistical analyses were performed using SPSS 17.0 (SPSS Inc., Chicago, IL). A Mann-Whitney test was used to detect differences in subgroup analysis. Receiver operating characteristic (ROC) curves served to develop the optimal cutoff point for serum hs-CRP levels concerning OS, PFS, LRFS, DMFS (S1 Fig). The score localized closest to the point at both maximum sensitivity and specificity was selected as the cutoff score leading to the greatest number of cancers that were correctly classified as having or not having the outcome. Here, hs-CRP cutoff points for OS, PFS, LRFS, and DMFS were 1.96 mg/L, 1.96 mg/L, 3.23 mg/L, and 1.96 mg/L, respectively. We then selected 1.96 mg/L as the hs-CRP cutoff point in our study. The Kaplan-Meier method was employed to estimate the cumulative survival plot. The survival between groups (hs-CRP>1.96 mg/L vs. hs-CRP≤1.96 mg/L) was compared using the log-rank test. Multivariate analysis was achieved using a Cox proportional hazards model, excluding insignificant variables by backward elimination. Factors that might have an impact on outcomes, such as age (>45 years vs. ≤45 years), sex (male vs. female), histology (Ⅲ vs.Ⅱ vs.Ⅰ), tumor stage (T4 vs. T3 vs. T2 vs. T1), node stage (N3 vs. N2 vs. N1 vs. N0), treatment method (chemoradiotherapy vs. radiotherapy), BMI (≥23 kg/m2 vs. <23 kg/m2), smoking status (ever and current vs. never), chronic HBV infection (yes vs. no), diabetes mellitus (yes vs. no), cardiovascular disease (yes vs. no), family history of NPC (yes vs. no), EBV DNA (>4000 copies/ml vs. ≤4000 copies/ml), VCA-IgA (>1:80 vs. ≤1:80), and EA-IgA (>1:10 vs. ≤1:10), were all adjusted in a Cox proportional hazards model. All reported probability values were two tailed, and P<0.05 was considered statistically significant.

## Results

### Patient characteristics

The characteristics of the 1,589 NPC patients are listed in Table 1. In total, there were 252 (15.9%) early stage (Ⅰ-Ⅱ) NPC patients and 1,337 (84.1%) advanced stage (Ⅲ-Ⅳ) patients in the cohort. All patients were treated according to the principles of treatment for NPC patients at Sun Yat-sen University Cancer Center, Guangzhou, China. A stratified, multi-therapeutic protocol was used. In all, radiation alone was administered for 235 (14.8%) patients, and radiation with concurrent platinum-based chemotherapy was administered for 648 (40.8%) individuals. 484 (30.5%) patients received concurrent chemoradiotherapy with neoadjuvant and 156 (9.8%) were treated with radiation plus neoadjuvant. Moreover, 52 (3.3%) received concurrent chemoradiotherapy with adjuvant and 14 (0.9%) completed concurrent chemoradiotherapy with both neoadjuvant and adjuvant. Neoadjuvant or adjuvant chemotherapy consisting of cisplatin plus 5-fluorouracil or cisplatin plus taxane was administered every 3 weeks for 2 or 3 cycles [27]. Concurrent cisplatin chemotherapy was administered on weeks 1, 4, and 7 of RT.

**Table 1:** Patient Characteristics.

**Characteristics**	**n(%)**	**hs-CRP, mg/L**		**P value**
		**≤1.96**	**>1.96**	
**Age(y)**				
**≤45**	828 (52.1)	518	310	0.417
**>45**	761 (47.9)	461	300	
**Sex**				
**Male**	1162 (26.9)	681	481	<0.001
**Female**	427 (73.1)	298	129	
**Histology, WHO type**				
**Ⅰ**	2 (0.1)	1	1	0.751^c^
**Ⅱ**	78 (4.9)	51	27	
**Ⅲ**	1509 (95.0)	927	582	
**Clinical stage** ^**a**^				
**Ⅰ**	65 (4.1)	57	8	<0.001
**Ⅱ**	187 (11.8)	138	49	
**Ⅲ**	870 (54.8)	555	315	
**ⅣA-B**	467 (29.4)	229	238	
**Tumor stage** ^**a**^				
**T1**	140 (8.8)	109	31	<0.001
**T2**	312 (19.6)	229	83	
**T3**	763 (48.0)	472	291	
**T4**	374 (23.5)	169	205	
**Node stage** ^**a**^				
**N0**	307 (19.3)	201	106	0.033
**N1**	548 (34.5)	353	195	
**N2**	610 (38.4)	349	261	
**N3**	124 (7.8)	76	48	
**Treatment**				
**Radiotherapy**	235 (14.8)	173	62	<0.001
**Chemoradiotherapy**	1354 (85.2)	806	548	
**Body mass index** ^**b**^, **kg/m** ^**2**^				
**<18.5**	123(7.7)	81	42	<0.001
**18.5–22.9**	678(42.7)	446	232	
**23.0–27.4**	663(41.7)	398	265	
**≥27.5**	125(7.9)	54	71	
**Smoking status**				
**Never**	999(62.9)	647	352	0.001
**Ever or current**	590(37.1)	332	258	
**Chronic HBV infection**				
**No**	1458(91.8)	898	560	0.957
**Yes**	131(8.2)	81	50	
**Diabetes mellitus**				
**No**	1548(97.4)	954	594	0.932
**Yes**	41(2.6)	25	16	
**Cardiovascular disease**				
**No**	1494(94.0)	937	557	<0.001
**Yes**	95(6.0)	42	53	
**Family history of NPC**				
**No**	1395(87.8)	853	542	0.308
**Yes**	194(12.2)	126	68	
**EBV DNA, copies/ml**				
**≤4000**	894(56.3)	596	298	<0.001
**>4000**	695(43.7)	383	312	
**VCA-IgA**				
**≤1:80**	408(25.7)	263	145	0.170
**>1:80**	1181(74.3)	716	465	
**EA-IgA**				
**≤1:10**	658(41.4)	418	240	0.187
**>1:10**	931(58.6)	561	370	
**Total**	1589(100%)	979(61.6)	610(38.4)	

Abbreviations: WHO = World Health Organization; HBV = hepatitis B virus; NPC = nasopharyngeal carcinoma; VCA = viral capsid antigen; EA = early antigen; Deaths = deceased patients at the last follow-up; Non-Deaths = patients alive at the last follow-up. PR = patients who progressed at the last follow-up; Non-PR = patients who had not progressed at the last follow-up; DM = patients presenting with distant metastasis at the last follow-up; Non-DM = patients without distant metastasis at the last follow-up. LR = patients presenting with local or regional relapse at the last follow-up; Non-LR = patients without local or regional relapse at the last follow-up.

P value was calculated with Pearson χ^2^ Test

^a^According to American Joint Committee on Cancer, 7^th^ edition.

^b^Different intervals for body mass index were divided according to the World Health Organization classifications for Asian populations.

^c^using Fisher’s Exact Test.

The median follow-up time was 44 months (IQR: 38–57). To the last date of follow-up, 67 of the 979 patients in the low hs-CRP (≤1.96 mg/L) group (6.8%) and 86 of the 610 patients in the high hs-CRP (>1.96 mg/L) group (14.1%) were dead. Worse outcomes, such as cancer progression (22.5% vs. 13.6%; p<0.001) and distant metastasis (17.5% vs. 8.4%; p<0.001) could be observed in the high hs-CRP group compared with low hs-CRP group.

**Fig 1:**
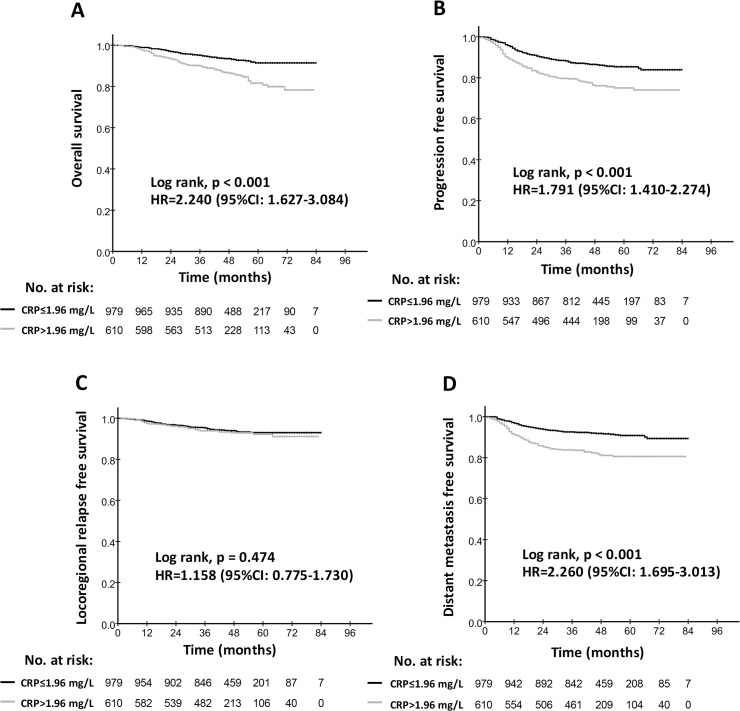
Kaplan-Meier survival curves for the low hs-CRP group (≤1.96 mg/L) and the high hs-CRP group (>1.96 mg/L) in the study population. Overall survival rates (A), progression-free survival rates (B), locoregional relapse-free survival (C) and distant metastasis-free survival rates (D) were compared in the entire cohort of NPC patients. Hazards ratios (HRs) were calculated using the unadjusted Cox proportional hazards model. P values were calculated using the unadjusted log-rank test.

We also found that patients with high hs-CRP levels displayed worse 4-year OS (86.4% vs. 93.5%; p<0.001), PFS (76.1% vs. 86.5%; p<0.001) and DMFS (81.1% vs. 91.9%; p<0.001) but not worse LRFS than did patients with low hs-CRP levels (Fig 1). Additionally, the 4-year cancer-specific survival rates were 86.9% and 94.1% for the high hs-CRP cohort and the low hs-CRP cohort, respectively (p<0.001).

### The relationship between baseline hs-CRP and other risk factors

As presented in detail in Table 1, the pretreatment serum hs-CRP level was correlated with sex, clinical stage, body mass index, smoking status, and EBV DNA level. Pretreatment serum hs-CRP levels for patients with advanced stage (median: 1.39 mg/L; IQR: 0.64–3.50 mg/L) NPC were significantly higher than those for early stage (median: 0.86 mg/L; IQR: 0.42–1.83 mg/L) patients (p<0.001). Compared to patients with EBV DNA ≤ 4000 copies/ml (median: 1.08 mg/L; IQR: 0.53–2.56 mg/L), those who had EBV DNA > 4000 copies/ml (median: 1.63 mg/L; IQR: 0.74–4.20 mg/L) were inclined to present with high hs-CRP levels (p<0.001).

In addition, significant differences in hs-CRP levels could be found between the groups with (median: 1.63 mg/L; IQR: 0.70–4.00 mg/L) and without (median: 1.09 mg/L; IQR: 0.56–2.84 mg/L) smoking history (p<0.001) and those with (median: 2.27 mg/L; IQR: 0.90–5.31 mg/L) and without (median: 1.22 mg/L; IQR: 0.59–3.01 mg/L) cardiovascular disease (p<0.001). However, no significant differences were detected between the groups with and without chronic HBV infection or diabetes mellitus.

### Univariate and multivariate analyses showed that hs-CRP was a predictor of clinical outcomes

Univariate analysis indicated that in addition to elevated hs-CRP levels, age, sex, TNM classification, smoking status, concurrent cardiovascular disease, high EBVDNA, and treatment allocation were significantly associated with worse survival for NPC patients (S1 Table).

In a multivariate analysis, after adjusting for other risk factors, hs-CRP was still an independent prognostic factor for OS (P = 0.001), PFS (P<0.001) and DMFS (P<0.001). Higher levels of hs-CRP predicted worse OS (HR: 1.723; 95%CI:1.238–2.398; p = 0.001), PFS (HR: 1.621; 95%CI: 1.273–2.064) and DMFS (HR: 1.879; 95%CI: 1.394–2.531) (Table 2).

**Table 2:** Multivariate analysis of prognostic factors for NPC patients.

					**95% CI for HR**	
**Outcomes**	**Number of events (%)**	**Variable**	**p**	**HR**	**lower**	**upper**
**OS**	**153 (9.6)**					
		**Age**	0.003	1.666	1.189	2.334
		**Sex**	0.057	1.485	0.988	2.230
		**Tumor stage** ^**a**^	0.008	1.346	1.082	1.675
		**Node stage** ^**a**^	0.000	1.448	1.182	1.772
		**BMI** ^**b**^	0.000	0.529	0.378	0.740
		**Cardiovascular disease**	0.060	1.660	0.979	2.814
		**EBV DNA**	0.000	2.841	1.938	4.164
		**hs-CRP**	**0.001**	**1.723**	1.238	2.398
**PFS**	**270 (17.0)**					
		**Sex**	0.046	1.342	1.006	1.789
		**Node stage** ^**a**^	0.000	1.325	1.142	1.538
		**BMI** ^**b**^	0.000	0.515	0.401	0.662
		**EBV DNA**	0.000	3.147	2.382	4.159
		**hs-CRP**	**0.000**	**1.621**	1.273	2.064
**LRFS**	**99 (6.2)**					
		**BMI** ^**b**^	0.005	0.559	0.371	0.842
		**EBV DNA**	0.000	2.550	1.689	3.852
**DMFS**	**189 (11.9)**					
		**Sex**	0.014	1.574	1.095	2.262
		**Tumor stage** ^**a**^	0.068	1.189	0.987	1.433
		**Node stage** ^**a**^	0.000	1.555	1.292	1.872
		**BMI** ^**b**^	0.000	0.562	0.418	.756
		**EBV DNA**	0.000	3.049	2.163	4.299
		**hs-CRP**	**0.000**	**1.879**	1.394	2.531

Abbreviations: CI = confident interval; HR = hazard ratio; OS = overall survival; BMI = body mass index; LRFS = locoregional relapse free survival; DMFS = distant metastasis free survival; other abbreviations are the same as Table 1.

A Cox proportional hazards regression model was used to detect variables one by one without adjustment. All variables were transformed into category variables. HRs were calculated for age (>45 years vs. ≤45 years), sex (male vs. female), histology (Ⅲ vs.Ⅱ vs.Ⅰ), tumor stage (T4 vs.T3 vs.T2 vs.T1), node stage (N3 vs. N2 vs. N1 vs. N0), treatment method (chemoradiotherapy vs. radiotherapy), BMI (≥23 kg/m2 vs. <23 kg/m2), smoking status (ever and current vs. never), Chronic hepatitis B (yes vs. no), diabetes mellitus (yes vs. no), cardiovascular disease (yes vs. no), family history of NPC (yes vs. no), EBV DNA (>4000 copies/ml vs. ≤4000 copies/ml), VCA-IgA (>1:80 vs. ≤1:80), EA-IgA (>1:10 vs. ≤1:10) and hs-CRP (>1.96 mg/L vs. ≤1.96 mg/L).

^a^According to American Joint Committee on Cancer, 7^th^ edition

^b^According to the World Health Organization classifications for Asian populations

### Prognostic significance of the hs-CRP within the UICC TNM classification and EBV DNA subgroup

**Fig 2:**
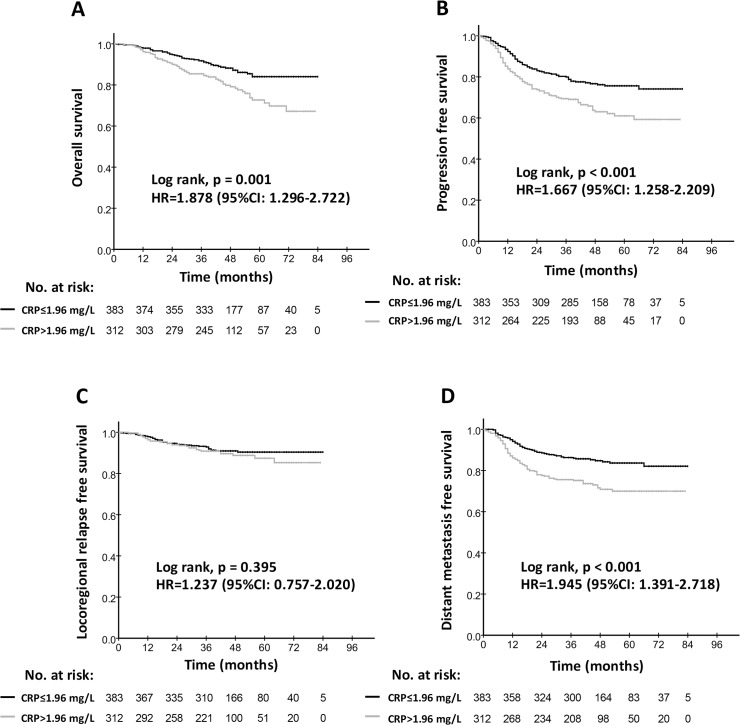
Kaplan-Meier survival curves for the low hs-CRP group (≤1.96 mg/L) and the high hs-CRP group (>1.96 mg/L) in patients with high EBV DNA. Overall survival rates (A), progression-free survival rates (B), locoregional relapse-free survival (C) and distant metastasis-free survival rates (D) were compared in the subgroup of NPC patients with EBV DNA>4000 copies/ml. Hazards ratios (HRs) were calculated using the unadjusted Cox proportional hazards model. P values were calculated using the unadjusted log-rank test.

As shown in Table 3, when 4-year survival rates were calculated in specific subgroups, statistical significance was detected especially for the advanced-stage (Ⅲ-Ⅳ) group, the low EBV DNA (≤4000 copies) group and the high EBV DNA (>4000copies) group (Fig 2), but not for patients with early-stage (Ⅰ-Ⅱ) disease.

**Table 3:** Four-year survival rate for patients in specific subgroups.

**Outcomes**	**Subgroups**	**Four-year survival rate (95%CI), %**				**P value**
		**hs-CRP≤1.96mg/L**		**hs-CRP >1.96mg/L**		
**OS**						
	**Clinical stageⅠ-Ⅱ** ^**a**^	**97.9**	(95.9–99.9)	**93.4**	(85.8–100.0)	0.289
	**Clinical stageⅢ-Ⅳ** ^**a**^	**92.4**	(90.4–94.4)	**85.7**	(82.6–88.8)	**<0.001**
	**Low EBV DNA**	**96.8**	(95.4–98.2)	**94.1**	(91.4–96.8)	**0.019**
	**High EBV DNA**	**88.2**	(84.7–91.7)	**79.1**	(74.0–84.2)	**0.001**
	**Chronic HBV**	**94.9**	(89.0–100.0)	**87.4**	(78.0–96.8)	**0.023**
	**CVD**	**90.2**	(81.0–99.4)	**79.1**	(68.1–90.1)	0.127
**PFS**						
	**Clinical stageⅠ-Ⅱ** ^**a**^	**93.9**	(90.4–97.4)	**87.2**	(78.4–96.0)	0.091
	**Clinical stageⅢ-Ⅳ** ^**a**^	**84.7**	(82.2–87.2)	**75.0**	(71.1–78.9)	**<0.001**
	**Low EBV DNA**	**92.8**	(90.6–95.0)	**89.8**	(86.3–93.3)	0.210
	**High EBV DNA**	**76.7**	(72.4–81.0)	**63.0**	(57.1–68.9)	**<0.001**
	**Chronic HBV**	**90.8**	(84.3–97.3)	**65.2**	(50.1–80.3)	**<0.001**
	**CVD**	**92.7**	(84.7–100.0)	**69.9**	(56.6–83.2)	**0.020**
**LRFS**						
	**Clinical stageⅠ-Ⅱ** ^**a**^	**96.6**	(88.6–100.0)	**90.8**	(83.2–98.4)	0.083
	**Clinical stageⅢ-Ⅳ** ^**a**^	**93.2**	(91.4–95.0)	**93.1**	(90.7–95.5)	0.969
	**Low EBV DNA**	**95.7**	(93.9–97.5)	**96.8**	(94.8–98.8)	0.408
	**High EBV DNA**	**91.0**	(88.1–93.9)	**88.8**	(84.7–92.9)	0.395
	**Chronic HBV**	**96.1**	(91.8–100.0)	**93.5**	(86.4–100.0)	0.346
	**CVD**	**93.7**	(84.9–100.0)	**94.0**	(87.3–100.0)	0.743
**DMFS**						
	**Clinical stageⅠ-Ⅱ** ^**a**^	**96.9**	(94.4–99.4)	**94.6**	(88.7–100.0)	0.527
	**Clinical stageⅢ-Ⅳ** ^**a**^	**90.6**	(88.4–92.8)	**79.7**	(76.0–83.4)	**<0.001**
	**Low EBV DNA**	**96.3**	(94.7–97.9)	**91.8**	(88.5–95.1)	**0.015**
	**High EBV DNA**	**84.8**	(81.1–88.5)	**70.9**	(65.4–76.4)	**<0.001**
	**Chronic HBV**	**95.0**	(90.1–99.9)	**67.6**	(52.9–82.3)	**<0.001**
	**CVD**	**97.6**	(92.9–100.0)	**71.4**	(58.1–84.7)	**0.002**

Abbreviations: CI = confident interval; HR = hazard ratio; OS = overall survival; CVD = cardiovascular disease; LRFS = locoregional relapse free survival; DMFS = distant metastasis free survival; other abbreviations are the same as Table 1.

Low EBV DNA means EBV DNA ≤4000 copies/ml, and high EBV DNA is EBV DNA>4000 copies/ml.

P value is calculated using log-rank method

^a^According to American Joint Committee on Cancer, 7^th^ edition

These results indicated that hs-CRP levels might play a complementary role to TNM stage and EBV DNA levels, facilitating more accurate prognostic stratification of NPC patients.

### Prognostic significance of hs-CRP for patients with chronic HBV infection and cardiovascular disease

**Fig 3:**
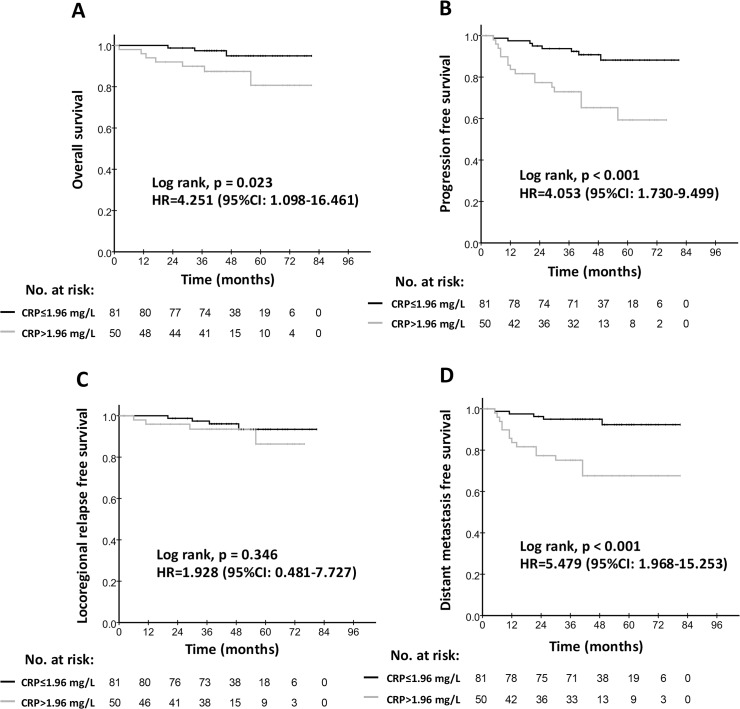
Kaplan-Meier survival curves for the low hs-CRP group (≤1.96 mg/L) and the high hs-CRP group (>1.96 mg/L) in patients with chronic HBV infection. Overall survival rates (A), progression-free survival rates (B), locoregional relapse-free survival (C) and distant metastasis-free survival rates (D) were compared in the subgroup of NPC patients with chronic HBV infection. Hazards ratios (HRs) were calculated using the unadjusted Cox proportional hazards model. P values were calculated using the unadjusted log-rank test.

**Fig 4:**
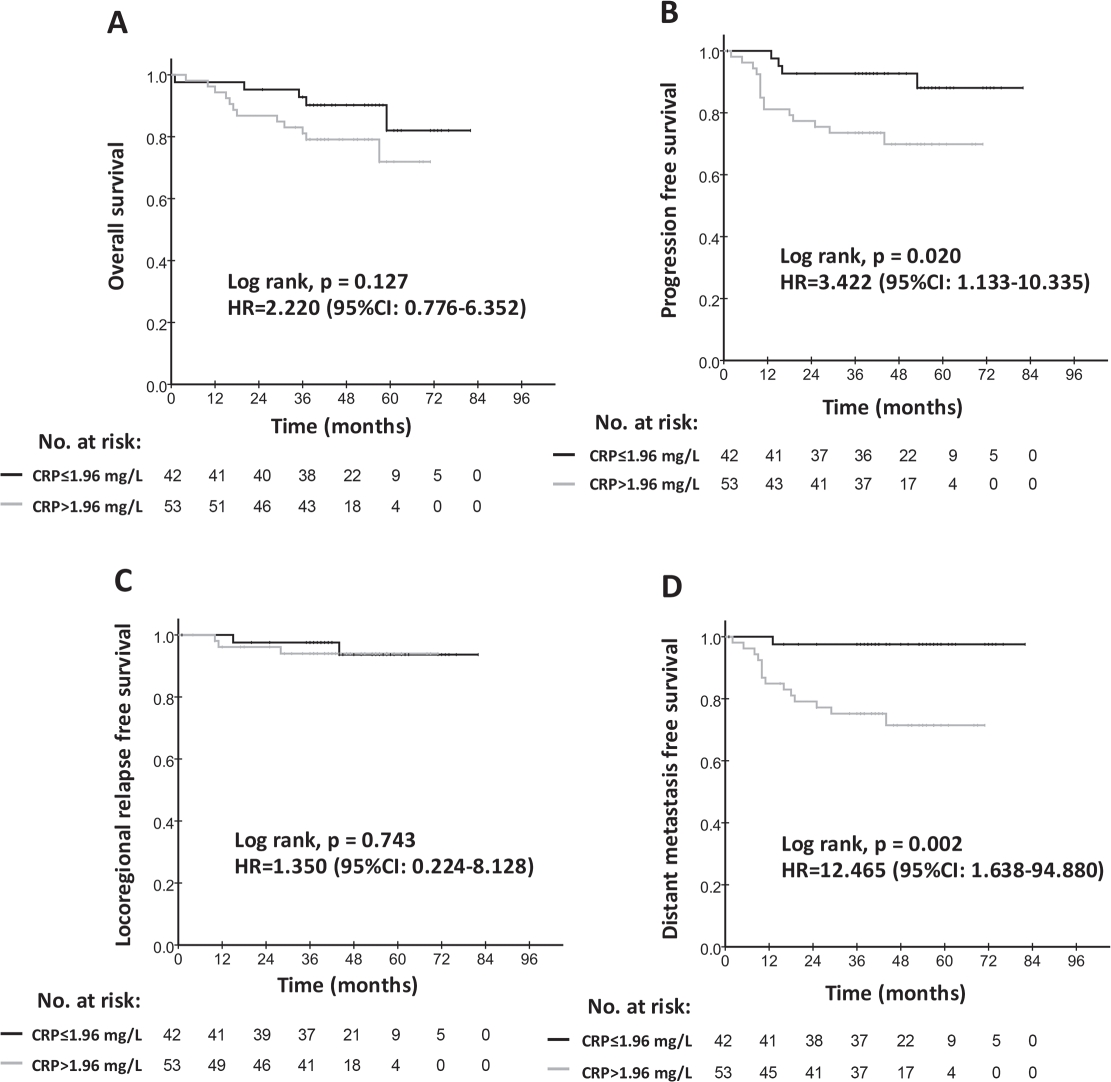
Kaplan-Meier survival curves for the low hs-CRP group (≤1.96 mg/L) and the high hs-CRP group (>1.96 mg/L) in patients with cardiovascular disease. Overall survival rates (A), progression-free survival rates (B), locoregional relapse-free survival (C) and distant metastasis-free survival rates (D) were compared in the subgroup of NPC patients with cardiovascular disease. Hazards ratios (HRs) were calculated using the unadjusted Cox proportional hazards model. P values were calculated using the unadjusted log-rank test.

We evaluated whether hs-CRP is still an effective prognostic indicator for NPC patients with cardiovascular disease and chronic hepatitis. Interestingly, we found that hs-CRP levels were good at survival prediction for the NPC patients with chronic HBV infection. Compared with patients with low hs-CRP levels, decreased survival was detected in the cohort of high hs-CRP, with 87.4% vs. 94.9% (p = 0.023) for OS, 65.2% vs. 90.8% (p<0.001) for PFS, and 67.6% vs. 95.0% (p<0.001) for DMFS (Table 3 and Fig 3). A similar finding was observed for patients with cardiovascular disease, with 79.1% vs. 90.2% (p = 0.020) for PFS, and 71.4% vs. 97.6% (p = 0.002) for DMFS (Table 3 and Fig 4).

## Discussion

With an observational study of 1,589 NPC patients in the IMRT era, elevated hs-CRP levels were correlated with worse OS, PFS and DMFS but not worse LRFS. Higher levels of pretreatment hs-CRP predicted poorer prognosis, playing a complementary role to TNM stage and plasma EBV DNA levels.

Hs-CRP, as an acute-phase protein, is a non-specific protein reacting to acute inflammation, infection, and tissue damage. When an acute-phase response happens, cytokines, predominantly IL-6, coming from the injured tissue will promote the synthesis of hs-CRP in the liver [20]. Epidemiological studies found that hs-CRP had a robust association with coronary heart disease (CHD) over the last decade [28]. In recent years, hs-CRP has become a popular biomarker for the prediction of clinical outcomes in cancer patients. High hs-CRP levels were considered to indicate an unfavorable prognosis for patients with colorectal cancer [11], osteosarcoma [12], hepatocellular carcinoma [13], prostate cancer [29] and renal cell carcinoma [30]. Recently, Xia reported that the combination of hs-CRP and N-classification could improve the prognostic stratification for NPC patients based on only two-dimensional conventional radiotherapy [15]. Currently, IMRT is widely used in clinical practice for NPC treatment, greatly contributing to reduced local and regional relapse. Interestingly, according to our findings and consistent with previous reports, our study confirmed that pretreatment serum hs-CRP is still an independent prognostic factor for NPC patients in the IMRT era.

Over the last decade, plasma EBV DNA levels have been found to be correlated with tumor burden [31], TNM stage [32], response to chemoradiotherapy [32–34], and survival in NPC patients [6, 7]. It is now a useful biomarker for the clinical management of NPC patients and is considered the most powerful biomarker to complement TNM stage [2]. Intriguingly, we found that pretreatment hs-CRP levels were complementary to EBV DNA levels to predict prognosis for NPC patients. These results indicate that EBV DNA levels alone are insufficient to complement the TNM classification. Most likely because of the biological heterogeneity of cancer, large variations in the clinical outcomes can be found in NPC patients with the same stage and similar treatment regimens, as well as even with the same EBV DNA level.

The mechanism to explain why the combination of these two biomarkers will improve the prognostic stratification of NPC patients is unclear. Previous studies demonstrated that EBV DNA molecules are released into the circulation by apoptosis and represent the tumor load [35, 36]. Tumor cells also express EBV-encoded LMP1, which was demonstrated to regulate the production of IL-6 in epithelial cells [37], and IL-6 is able to promote NPC progression [38] and increase hs-CRP levels [20]. These findings partially explain why EBV DNA levels may correlate with hs-CRP levels. However, the mechanism of elevated hs-CRP level promoted the progression for NPC patients still need to explore in future study.

Epidemiologists found that elevated CRP levels could be examined in patients with acute viral hepatitis [39], cardiovascular disease [17] and type 2 diabetes mellitus [40]. Consistent with these epidemiological discoveries, we found that hs-CRP levels were significantly higher in NPC patients with cardiovascular disease. Interestingly, we also found that the prognostic ability of hs-CRP was applicable to patients with cardiovascular disease when it came to PFS and DMFS.

It was reported that patients with chronic HBV infection represented worse outcomes [41]. Immunological dysfunction was considered the latent mechanism leading to an unfavorable prognosis of HBV-infected NPC patients in that report. Despise the presence of hepatitis, hs-CRP still acted as a reliable indicator for prognosis. However, the potential interaction between hs-CRP and concurrent disease, such as cardiovascular disease and chronic HBV infection, should be further explored.

The major disadvantage of our study is the single measurement of serum hs-CRP recorded from one single center. Although our cancer center treats a large number of NPC patients, these results need to be validated in other data sets. Another limitation is that the median follow-up time is 44 months, and patients must remain closely followed up and report 5-year follow-up results as available.

In conclusion, our study demonstrated that baseline serum hs-CRP levels were an independent prognostic factor for NPC patients treated with IMRT and the subgroup patients with cardiovascular disease and chronic HBV infection. Because the measurement of hs-CRP is established, a routinely measured blood-based parameter that is reproducibly detected without additional laborious efforts before use in clinical applications, we believe that hs-CRP could have a promising application in NPC management. In the future, we look forward to developing a nomogram combining plasma EBV DNA, hs-CRP, tumor host and facility related factors and clinical staging to identify high risk patients of relapse and more individualized treatment strategies would be catered according to these factors.

## Supporting Information

S1 Dataset Original data used for all statistical analyses in this article (SAV)

S1 Fig Receiver operating characteristic (ROC) curves for hs-CRP. Pretreatment serum hs-CRP serves as a predictor of (A) death, (B) progression, (C) locoregional relapse, (D) distant metastasis. The area under the ROC curve (AUC) was calculated for each graph.
(TIF)

S1 Table Univariate analysis of prognostic factors for NPC patients. (DOC)
