# Supplementary figures and images for: Elevated High-Sensitivity C-Reactive Protein Levels Predict Decreased Survival for Nasopharyngeal Carcinoma Patients in the Intensity-Modulated Radiotherapy Era
Source: PLoS One. 2015 Apr 13;10(4):e0122965. doi: 10.1371/journal.pone.0122965 (PMC4395211; doi:10.1371/journal.pone.0122965)

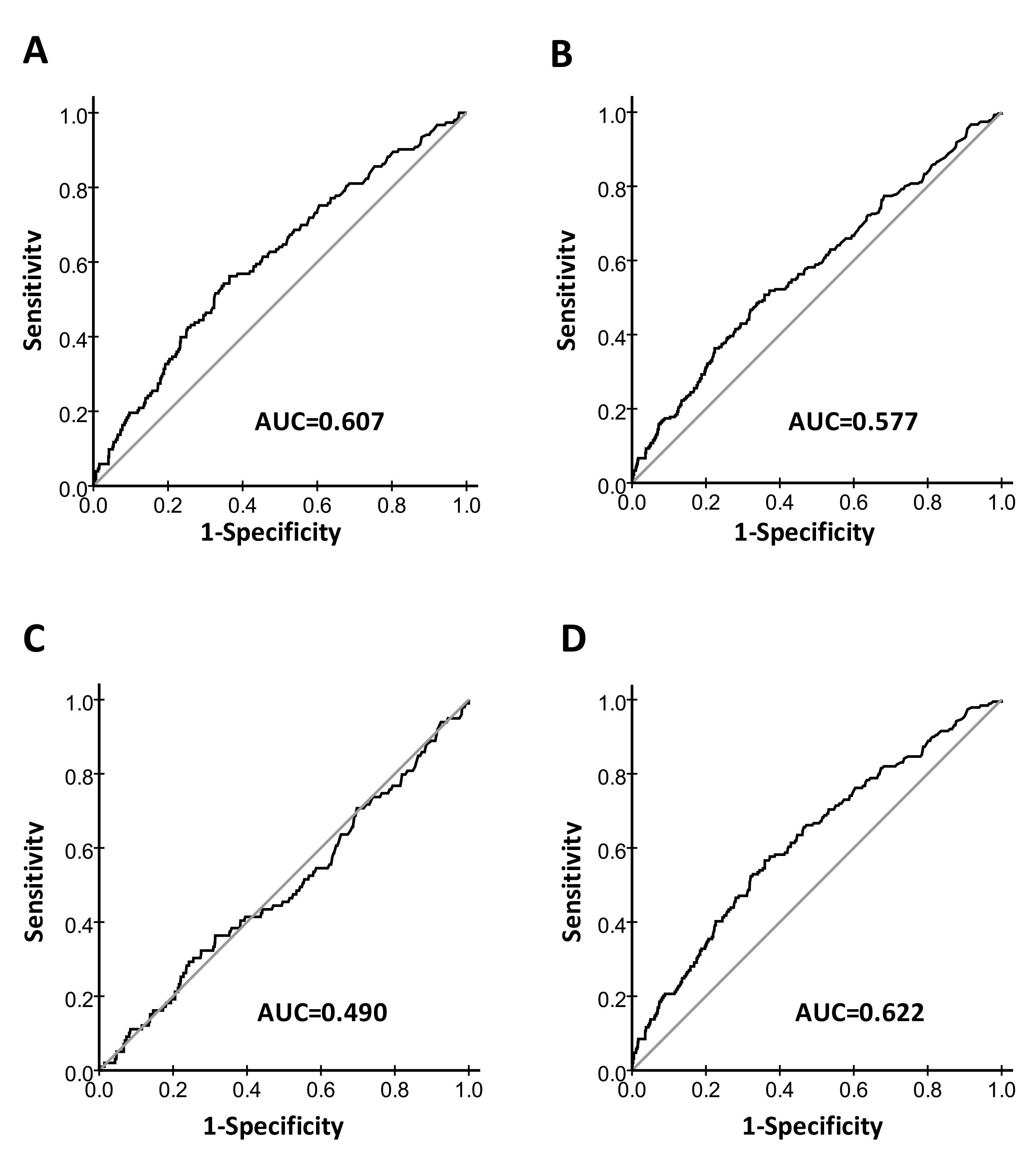

Supplement: S1 Fig — Pretreatment serum hs-CRP serves as a predictor of (A) death, (B) progression, (C) locoregional relapse, (D) distant metastasis. The area under the ROC curve (AUC) was calculated for each graph. (TIF) [file pone.0122965.s002.tif]
