# Supplementary material for: Elevated High-Sensitivity C-Reactive Protein Levels Predict Decreased Survival for Nasopharyngeal Carcinoma Patients in the Intensity-Modulated Radiotherapy Era
Source: PLoS One. 2015 Apr 13;10(4):e0122965. doi: 10.1371/journal.pone.0122965 (PMC4395211; doi:10.1371/journal.pone.0122965)
Supplement: S1 Table — (DOC) [file pone.0122965.s003.doc]

**S1 Table. Univariate** analysis of prognostic factors for NPC patients

|  | |  |  |  |  | **95%CI for HR** | |
| --- | --- | --- | --- | --- | --- | --- | --- |
| **Outcomes** | | **Variable** | **B** | **p** | **HR** | **lower** | **upper** |
| **OS** |  | |  |  |  |  |  |
|  | **Age** | | 0.568 | 0.001 | 1.764 | 1.274 | 2.443 |
|  | **Sex** | | 0.431 | 0.034 | 1.539 | 1.033 | 2.294 |
|  | **Histology** | | 0.078 | 0.838 | 1.081 | 0.511 | 2.287 |
|  | **Tumor stage^a^** | | 0.512 | 0.000 | 1.668 | 1.352 | 2.058 |
|  | **Node stage^a^** | | 0.479 | 0.000 | 1.615 | 1.339 | 1.947 |
|  | **Treatment** | | 0.719 | 0.017 | 2.053 | 1.139 | 3.702 |
|  | **BMI^b^** | | -0.479 | 0.004 | 0.620 | 0.447 | 0.858 |
|  | **Smoking status** | | 0.455 | 0.005 | 1.576 | 1.147 | 2.164 |
|  | **Chronic HBV infection** | | -0.230 | 0.481 | 0.794 | 0.418 | 1.508 |
|  | **Diabetes mellitus** | | -0.692 | 0.331 | 0.500 | 0.124 | 2.020 |
|  | **Cardiovascular disease** | | 0.726 | 0.005 | 2.066 | 1.248 | 3.421 |
|  | **Family history of NPC** | | -0.333 | 0.235 | 0.717 | 0.414 | 1.242 |
|  | **EBV DNA, copies/ml** | | 1.416 | 0.000 | 4.121 | 2.856 | 5.947 |
|  | **VCA-IgA** | | 0.174 | 0.365 | 1.190 | 0.816 | 1.736 |
|  | **EA-IgA** | | 0.061 | 0.710 | 1.063 | 0.770 | 1.468 |
|  | **hs-CRP** | | 0.807 | 0.000 | 2.240 | 1.627 | 3.084 |
| **PFS** |  | |  |  |  |  |  |
|  | **Age** | | 0.132 | 0.280 | 1.141 | 0.898 | 1.448 |
|  | **Sex** | | 0.220 | 0.126 | 1.247 | 0.940 | 1.653 |
|  | **Histology** | | -0.090 | 0.731 | 0.914 | 0.548 | 1.524 |
|  | **Tumor stage^a^** | | 0.281 | 0.000 | 1.324 | 1.144 | 1.532 |
|  | **Node stage^a^** | | 0.462 | 0.000 | 1.588 | 1.380 | 1.826 |
|  | **Treatment** | | 0.661 | 0.002 | 1.936 | 1.263 | 2.969 |
|  | **BMI^b^** | | -0.569 | 0.000 | 0.566 | 0.442 | 0.725 |
|  | **Smoking status** | | 0.218 | 0.078 | 1.244 | 0.976 | 1.584 |
|  | **Chronic HBV infection** | | 0.098 | 0.646 | 1.103 | 0.726 | 1.678 |
|  | **Diabetes mellitus** | | -0.358 | 0.427 | 0.699 | 0.289 | 1.693 |
|  | **Cardiovascular disease** | | 0.207 | 0.384 | 1.230 | 0.772 | 1.961 |
|  | **Family history of NPC** | | -0.085 | 0.655 | 0.918 | 0.632 | 1.335 |
|  | **EBV DNA, copies/ml** | | 1.348 | 0.000 | 3.849 | 2.945 | 5.030 |
|  | **VCA-IgA** | | 0.214 | 0.144 | 1.238 | 0.929 | 1.650 |
|  | **EA-IgA** | | 0.161 | 0.199 | 1.175 | 0.919 | 1.502 |
|  | **hs-CRP** | | 0.583 | 0.000 | 1.791 | 1.410 | 2.274 |
| **LRFS** |  | |  |  |  |  |  |
|  | **Age** | | 0.000 | 0.997 | 0.999 | 0.674 | 1.483 |
|  | **Sex** | | 0.059 | 0.797 | 1.061 | 0.678 | 1.659 |
|  | **Tumor stage^a^** | | 0.089 | 0.448 | 1.093 | 0.868 | 1.377 |
|  | **Node stage^a^** | | 0.149 | 0.192 | 1.161 | 0.928 | 1.454 |
|  | **Treatment** | | 0.347 | 0.278 | 1.414 | 0.756 | 2.647 |
|  | **BMI^b^** | | -0.583 | 0.005 | 0.558 | 0.371 | 0.841 |
|  | **Smoking status** | | 0.169 | 0.409 | 1.184 | 0.793 | 1.769 |
|  | **Chronic HBV infection** | | -0.015 | 0.968 | 0.985 | 0.478 | 2.030 |
|  | **Diabetes mellitus** | | -0.264 | 0.712 | 0.768 | 0.189 | 3.116 |
|  | **Cardiovascular disease** | | -0.154 | 0.737 | 0.857 | 0.349 | 2.107 |
|  | **Family history of NPC** | | 0.073 | 0.807 | 1.075 | 0.600 | 1.927 |
|  | **EBV DNA, copies/ml** | | 0.937 | 0.000 | 2.553 | 1.691 | 3.856 |
|  | **VCA-IgA** | | 0.269 | 0.274 | 1.308 | 0.808 | 2.119 |
|  | **EA-IgA** | | 0.191 | 0.359 | 1.210 | 0.805 | 1.818 |
|  | **hs-CRP** | | 0.147 | 0.475 | 1.158 | 0.775 | 1.730 |
| **DMFS** |  | |  |  |  |  |  |
|  | **Age** | | 0.136 | 0.350 | 1.146 | 0.861 | 1.524 |
|  | **Sex** | | 0.407 | 0.025 | 1.502 | 1.052 | 2.143 |
|  | **Histology** | | -0.043 | 0.893 | 0.958 | 0.513 | 1.788 |
|  | **Tumor stage^a^** | | 0.379 | 0.000 | 1.461 | 1.221 | 1.749 |
|  | **Node stage^a^** | | 0.586 | 0.000 | 1.796 | 1.514 | 2.132 |
|  | **Treatment** | | 0.886 | 0.002 | 2.426 | 1.381 | 4.261 |
|  | **BMI^b^** | | -0.465 | 0.002 | 0.628 | 0.469 | 0.841 |
|  | **Smoking status** | | 0.246 | 0.094 | 1.279 | 0.959 | 1.707 |
|  | **Chronic HBV infection** | | 0.245 | 0.311 | 1.278 | 0.795 | 2.053 |
|  | **Diabetes mellitus** | | -0.213 | 0.673 | 0.808 | 0.300 | 2.176 |
|  | **Cardiovascular disease** | | 0.343 | 0.202 | 1.409 | 0.832 | 2.388 |
|  | **Family history of NPC** | | -0.240 | 0.322 | 0.787 | 0.490 | 1.264 |
|  | **EBV DNA, copies/ml** | | 1.452 | 0.000 | 4.274 | 3.073 | 5.944 |
|  | **VCA-IgA** | | 0.117 | 0.495 | 1.124 | 0.804 | 1.570 |
|  | **EA-IgA** | | 0.045 | 0.759 | 1.046 | 0.783 | 1.399 |
|  | **hs-CRP** | | 0.815 | 0.000 | 2.260 | 1.695 | 3.013 |

Abbreviations: CI = confident interval; HR= hazard ratio; OS = overall survival; BMI = body mass index; LRFS = locoregional relapse free survival; DMFS = distant metastasis free survival; other abbreviations are the same as table 1.

Cox proportional hazards regression model was used to detect variables one by one without adjustment. All variables were transformed into category variables. HRs were calculated for age (>45 years vs. ≤45 years), sex (male vs. female), histology (Ⅲ vs.Ⅱ vs.Ⅰ), tumor stage (T4 vs.T3 vs.T2 vs.T1), node stage (N3 vs. N2 vs. N1 vs. N0), treatment method (chemoradiotherapy vs. radiotherapy), BMI (≥23 kg/m2 vs. <23 kg/m2), smoking status (ever and current vs. Never), Chronic hepatitis B (yes vs. no), diabetes mellitus (yes vs. no), cardiovascular disease (yes vs. no), family history of NPC (yes vs. no), EBV DNA (>4000 copies/ml vs. ≤4000 copies/ml), VCA-IgA (>1:80 vs. ≤1:80), EA-IgA (>1:10 vs. ≤1:10) and hs-CRP (>1.96 mg/L vs. ≤1.96 mg/L).

^a^According to American Joint Committee on Cancer, 7^th^ edition

^b^According to the World Health Organization classifications for Asian populations
